# The Global Burden of Disease survey 2010, *Lifting The Burden* and thinking outside-the-box on headache disorders

**DOI:** 10.1186/1129-2377-14-13

**Published:** 2013-02-15

**Authors:** Paolo Martelletti, Gretchen L Birbeck, Zaza Katsarava, Rigmor H Jensen, Lars J Stovner, Timothy J Steiner

**Affiliations:** 1 Department of Clinical and Molecular Medicine, Sapienza University, Rome, Italy; 2 Michigan State University, East Lansing, MI, USA; 3 Chikankata Hospital, Mazabuka, Zambia; 4 Department of Neurology, University of Essen, Essen, Germany; 5 Department of Neurology, University of Copenhagen, Copenhagen, Denmark; 6 Department of Neuroscience, Norwegian University of Science and Technology, Trondheim, Norway

## Editorial

The perception of a disease in the collective imaginations of people affected by it, of the general public, of health-care providers and of health policy-makers depends, undoubtedly, on a multiplicity of diverse factors: its effects on mortality, its consequences in terms of ill health and disability, its economic impact, its implications for health services and health-resource consumption, the availability of treatments, the potential for the development of new pharmacological agents and interest aroused by the media. Chronic diseases also raise the question of patients’ reduced productive capacity over a long period – perhaps a life-time.

Furthermore, it is evident that, in the face of concrete reductions in economic global resources, public health care must confront increasing demands. Increased expenditure on health cannot necessarily be expected; neither may it always lead to the anticipated or desired outcomes.

In the past decade, headache has acquired a social dimension never previously recognized. It has always been part of people’s daily lives, as a very prevalent symptom of a multitude of disorders (but only a few of public-health importance), but scarcely has it appeared of pressing importance or deserving of high priority. Therefore, if it has received attention at all, this has been scant and grudging. All this may have changed, through the years, for a series of reasons: a better knowledge has been gained of the mechanisms of headache, and has been followed by the invention, development and marketing of specific drugs at least for migraine; there are better academic initiatives to educate in the field; but, most of all, there is now a large, and growing, body of work to attest the societal impact of headache disorders, their epidemiology, their cost and the humanitarian necessity (and economic wisdom) of creating and supporting structured health care dedicated to their mitigation [1-7]. We see increasing requests for medical education in the field of headache [8]. Yet it cannot be said that other research in this field is going through a period of innovation: with a dearth of new ideas and decreasing resources, few large research groups and fewer pharmaceutical companies see headache as a fertile area for their endeavours.

The recent publication of the Global Burden of Disease survey 2010 (GBD 2010) [9] overturns all the prejudices, by demonstrating, with great clarity for those willing to see, where headache stands among the many causes of disability worldwide. Tension-type headache and migraine are the second and third most prevalent disorders in the world (after dental caries); migraine is the seventh highest cause of disability in the world. Is the world willing to see? Will it now take steps to remedy the past neglect of headache disorders and the inadequacies of its responses to them [7]? GBD 2010 is proof that it must.

We have the basis on which to start again, to rethink the approach to headache as a major factor in public ill-health, with huge implications for health care and great impact on the global economy [10]. Health policy is focusing more and more on chronic non-communicable diseases. This evidence of the high place among these of headache disorders must stimulate a new renaissance in academic clinical research and call for new approaches for industrial strategies, moving their interests from serendipity to true, planned innovation.

*Lifting The Burden*, through its unprecedented ten years of action, has traced a new way, working side by side with supranational organizations, with prestigious international academies, with Patients’ Associations and with many, many individuals. This long path has spread new ideas, created new structures from previously separated components, consolidated the actions – transforming them into synergies – of different partners with the same purpose: the control of headache disorders.

It is time to thank the partners in this network, this project which is the Global Campaign against Headache led by *Lifting The Burden*: the World Health Organization, The Journal of Headache and Pain, the Universities that have committed to it – the Norwegian University of Science and Technology in Trondheim and Sapienza University of Rome along with many other academic institution in the world – and a very large number of individual collaborators who cannot be listed here but many of whom are acknowledged at http://www.l-t-b.org/.

Out-of-the-box thinking has driven the Campaign, and made it a fly-wheel of incredible efficiency – and efficacy.

## Competing interests

All Authors are Directors and Trustees of *Lifting The Burden*, a UK-registered charity leading the Global Campaign against Headache in Official Relations with the World Health Organization.
